# Supplementary material for: Demographics, culture and participatory nature of multi-marathoning—An observational study highlighting issues with recommendations
Source: PLoS One. 2024 May 8;19(5):e0302602. doi: 10.1371/journal.pone.0302602 (PMC11078339; doi:10.1371/journal.pone.0302602)
Supplement: S2 File — (PDF) [file pone.0302602.s002.pdf]

Multi Marathon Survey

Continents What continent do you live in?

○ Asia (1)

○ Africa (2)

○ Europe (3)

○ North America (4)

○ South America (5)

○ Australia/Oceania (6)

*

Q36 What country are you resident?

____

Countries Which country do you live in?
Additional countries will be added here as the survey is rolled out

- ○ Denmark (8)
- ○ England (1)
- ○ Germany (7)
- ○ Italy (9)
- ○ Northern Ireland (2)
- ○ Scotland (3)
- ○ Wales (4)
- ○ Republic of Ireland (5)
- ○ Other European Country (6) ____

Gender To which gender identity do you most identify?

- ◯ Male (1)
- ◯ Female (2)
- ◯ Transgender Female (3)
- ◯ Trangender Male (4)
- ◯ Gender Variant/Non-Conforming (5)
- ◯ Not Listed (Please Specify) (6) ____
- ◯ Prefer not to say (7)

---

*

Age What age are you?
Please type in your age.

____

End of Block: Block 1 Basics

Start of Block: Block 2 Running History

Total Mar How many Marathons/Ultras have you completed?
Drag the bar below to the required number or Click radio button if over 1000,
Over 1000

	0	200	400	600	800	1000
Number of Marathons/Ultras completed (1)						

Percentage What is the percentage of Marathons vrs Ultras completed?
e.g. If you have completed 50 marathon/Ultra events and 40 were Marathons choose 80%

- ○ 0-20% (1)
- ○ 21-40% (2)
- ○ 41-60% (3)
- ○ 61-80% (4)
- ○ 81-100% (5)
- ○ Don't know (6)

PB What is your marathon PB/PR?
Use the drop down list to specify the range of your Personal Best/ Personal Record (PB/PR) time for completing a marathon

▼ 2:00:2:15 (1) ... >6:00 (17)

Running history Multi Marathon history
Drag the bar to the appropriate age. Use the age that you took up long distance running/jogging/walking with a view to completing marathon distance (rather than school cross country etc..)

	Age	Don't know
	0 10 20 30 40 50 60 70 80 90 100	
At what age did you start in the longer distance events? ()		
At what age did you complete your first marathon/Ultra? ()		

Annually On average how many Marathon/Ultra events do you complete annually?
Choose a range and click next button

- ◯ 0-10 (1)
- ◯ 11-20 (2)
- ◯ 21-30 (3)
- ◯ 31-40 (4)
- ◯ 41-50 (5)
- ◯ >51 (6)

End of Block: Block 2 Running History

Start of Block: Block 3 Motivations and Awards

Motivation What motivates you to complete marathons/ultras?
Click all applicable and hit Next button

- ☐ Awards (1)
- ☐ Competition (2)
- ☐ Continuous performance improvement (3)
- ☐ It is a way of life (4)
- ☐ Keeping fit and healthy (5)
- ☐ Multi Marahon Series (e.g. 10 in 10 days) (14)
- ☐ Running streaks (6)
- ☐ Sense of accomplishment (7)
- ☐ Social life (8)
- ☐ To reach certain milestones (9)
- ☐ Total numbers (10)
- ☐ Travel (11)
- ☐ Others - Please specify (12) ____
- ☐ ⊗None of the above (13)

Series Which Multi-marathon series do you participate in?

- ☐ 5 in 5 days (1)
- ☐ 10 in 10 days (2)
- ☐ 12 in 12 months (9)
- ☐ 52 in 52 weeks (3)
- ☐ 100 in 100 weeks (4)
- ☐ Back to Back (6)
- ☐ Other - Please specify (7) ________________
- ☐ ⊗ None of the above (8)

100 marathons imp How important was achieving 100 marathons/Ultras?
Click the number that represents the importance to you and click Next

- 0 (0)
- 1 (1)
- 2 (2)
- 3 (3)
- 4 (4)
- 5 (5)
- 6 (6)
- 7 (7)
- 8 (8)
- 9 (9)
- 10 (10)

Awards imp How important is receiving awards or being part of an award club?
Click the number that represents the importance to you and click Next

- ○ 0 (0)
- ○ 1 (1)
- ○ 2 (2)
- ○ 3 (3)
- ○ 4 (4)
- ○ 5 (5)
- ○ 6 (6)
- ○ 7 (7)
- ○ 8 (8)
- ○ 9 (9)
- ○ 10 (10)

Awards Which of the following award clubs do you participate in?
Click all options applicable and click Next

- ☐ 7 continents (1)
- ☐ 7 continents in 7 days (2)
- ☐ 50 US states (3)
- ☐ 100 Marathons from your national club (4)
- ☐ 100 Marathon club UK milestones (5)
- ☐ Abbot Marathon Majors 6 stars (6)
- ☐ Atlas 100-mile club milestones (7)
- ☐ Global Marathon challenges (8)
- ☐ Marathon Globetrotters (9)
- ☐ Marathon Maniacs (10)
- ☐ MCI Ireland club milestones (11)
- ☐ Mega Marathons (12)
- ☐ Triple crown of Ultras (Comrades, Western States, UTMB) (13)
- ☐ World Mega Marathon ranking 300+ (14)
- ☒ None of these (15)
- ☐ Other - Please specify (16) ____

End of Block: Block 3 Motivations and Awards

Start of Block: Block 4 DIET

Diet Do you follow any diet?
Click the option that represents your diet or enter custom diet in 'other' box

○ Paleo (1)

○ Pesco-vegetarian (includes fish) (2)

○ Regular (including foods from all food groups) (3)

○ Vegan (4)

○ Vegetarian (excludes fish, poultry, and meat) (5)

○ Other (Please specify) (6) ____

○ Prefer not to say (7)

End of Block: Block 4 DIET

Start of Block: Block 8 Pandemic

Covid Did you contract COVID?
Choose "Probably Yes" if you have not tested positve but think you have had COVID.

○ No (1)

○ Yes (positive test) (2)

○ Probably yes (no positive test) (3)

Symptons Did you experience negative long-term (>8 weeks) effects?
Please choose all relevant and click Next

- ▢ Elevated Heart rate (1)
- ▢ Forced to stop running for a period (2)
- ▢ Heart Palpitations (3)
- ▢ Muscle aches (4)
- ▢ Shortness of breath (5)
- ▢ Unusual fatigue (6)
- ▢ ⊗None (8)
- ▢ Other (Please specify) (7) ____

End of Block: Block 8 Pandemic

Start of Block: Block 5 Training and Equipment

*

kms How many kms do you exercise in a typical week?
Enter the no of kms your typically cover in an average week including events and training

____

Page Break

Multi Questions Please answer Yes or No to each question then click Next

	Yes (1)	No (2)
Do you follow a regular training regime from a coach or training plan? (1)	○	○
Do you do warmups before excercise? (7)	○	○
Are you a member of an organized traditional athletics club? (2)	○	○
Do you wear a GPS enabled device during training or events? (4)	○	○
Do you excercise with music/podcasts? (5)	○	○

HRZ Do you train or do events by Heartrate Zone (Zone 2 etc) ?

○ Yes (1)

○ No (2)

HR Strap Do you wear a Heart Rate Chest Strap?

○ Yes (1)

○ No (2)

Headphones Do you use bone conducting headphones?

- Yes (1)
- No (2)

Shoe Which type of shoe(s) do you normally use?
Select all applicable for both Road and Trail then click next

- Cushioned (1)
- Motion Control (made to support heavy weight, flat feet or severe overpronation) (2)
- Stability (3)
- Light weight (4)
- Minimal e.g. Vibram or flip flops (6)
- Other - Please specify (5) ____

End of Block: Block 5 Training and Equipment

Start of Block: Block 6 Fitness Stats

Stats Which commonly available fitness stats do you monitor?
Please click all applicable

- [ ] Calories (1)
- [ ] Heart Rate Variability (2)
- [ ] Floors (3)
- [ ] Intensity minutes (4)
- [ ] Max HR (5)
- [ ] Pulse Ox (6)
- [ ] Respiration (7)
- [ ] Resting HR (8)
- [ ] Sleep (9)
- [ ] Steps (10)
- [ ] Stress (11)
- [ ] VO2MAX (12)
- [ ] ⊗None (13)
- [ ] Other - Please Specify (14) ____

End of Block: Block 6 Fitness Stats

Start of Block: Block 7 Injuries and Recovery

Inj and Rec Have you had any of the following injuries?
Select all anwers that apply

- ☐ Ankle Injury (1)
- ☐ Blisters (2)
- ☐ Chafing (3)
- ☐ Calf Injury (4)
- ☐ Frostbite (5)
- ☐ Heat Exhaustion (6)
- ☐ Hip Injury (7)
- ☐ Hypothermia (8)
- ☐ IT Band Syndrome (9)
- ☐ Knee Injury (10)
- ☐ Muscle Pull (11)
- ☐ Morton's Neuroma (12)
- ☐ Plantar Fasciitis (13)
- ☐ Runners Knee (14)
- ☐ Shin Splints (15)
- ☐ Stress Fracture (16)
- ☐ Tendonitus (Achilles tendinopathy) (17)

- [ ] ⊗None (18)
- [ ] Other - Please specify (19)

Treatments Which treatments do you find most useful?
Click all applicable

- [ ] Accupuncture (1)
- [ ] Compression (2)
- [ ] Elevation (3)
- [ ] Foam Rolling (4)
- [ ] Ice (Ice packs or cold compresses) (5)
- [ ] Pain Relief (6)
- [ ] Rest (7)
- [ ] RICE (Rest Ice Compression Elevation) (8)
- [ ] Support strapping (9)
- [ ] Stretching (10)
- [ ] Surgery (11)
- [ ] ⊗None (13)
- [ ] Other - Please Specify (12)

Help Do you seek professional treatment for injuries?
If Yes then please specify what type of profressional you have seen?

- [ ] Physiotherapist (1)
- [ ] Another Medical Professional (e.g. Physician, A&E) (2)
- [ ] Specialist or other- Please detail here: (3) ____________________
- [x] No I do not seek professional advise for running injuries (4)

Multi questions 2 Please answer Y/N to the following questions

	Yes (1)	No (2)
Do you take medications that relieve pain before or during a marathon/Ultra? (1)	○	○
Has your doctor ever said that you have a heart condition? (2)	○	○
Has your doctor ever said that you have high blood pressure? (3)	○	○
Do you lose balance because of dizziness OR have you lost consciousness in the last 12 months? (4)	○	○
Do you currently have (or have had within the past 12 months) a bone, joint, or soft tissue (muscle, ligament, or tendon) problem that could be made worse by vigorous exercise? (5)	○	○
Do you see multi marathoning as positve for your mental health? (6)	○	○
Has your involvemement in multi marathoning been a contributory factor to life stressors? e.g. relationship breakdown, long term illness, Job loss (7)	○	○

Chronic Have you ever been diagnosed with another chronic medical condition (other than heart disease or high blood pressure)?
If Yes, Please list condition(s) here:

○ No (1)

○ Yes - Please specify (2) ____

Medication Are you currently taking prescribed medications for a chronic medical condition?
If Yes Please list condition(s) here:

○ No (1)

○ Yes - Please specify (2) ____
